# Control of media browning during micropropagation and assessment of biochemical and clonal fidelity of in vitro-derived and mother plants in *Thottea siliquosa* (Lamk.) Ding Hou., an important ethnomedicinal shrub

**DOI:** 10.1186/s43141-023-00523-8

**Published:** 2023-06-02

**Authors:** Chandran Padikkal Krishna Vrundha, Thuruthiyil Dennis Thomas

**Affiliations:** Department of Plant Science, Central University of Kerala, Tejaswini Hills, Periye (PO), Kasaragod, Kerala PIN-671320 India

**Keywords:** Callus, Conservation, Medicinal plant, Micropropagation, Organogenesis

## Abstract

**Background:**

*Thottea siliquosa* (Lamk.) Ding Hou., an important medicinal shrub, is widely used in both ayurvedic and indigenous systems of medicine. Root being the most useful part, the plant is constantly uprooted and thus puts pressure on the natural population. Until date, no micropropagation study is available in this plant. The objective of the study is to develop an efficient in vitro propagation protocol and assessment of clonal fidelity of *T. siliquosa*.

**Results:**

Media browning was a serious issue during micropropagation, and the addition of 40.0 mg/L ascorbic acid reduced the media browning. For direct shoot regeneration, the optimum response (92% frequency with 20.9 shoots per explant) was obtained when 7-day-old cotyledons were cultured on WPM supplemented with 1.0 mg/L thidiazuron and 0.25 mg/L α-naphthalene acetic acid. The cultures were transferred to WPM augmented with 0.4 mg/L thidiazuron for shoot elongation and growth. On this medium, 100% of cultures responded with a mean number of 27.6 shoots. For callus induction, MS medium with 1.0 mg/L 2,4-dichlorophenoxyacetic acid and 0.5 mg/L N_6_-benzylaminopurin was used. Shoot organogenesis was initiated on the same medium, and calli with minute shoots were transferred to MS medium fortified with 0.5 mg/L N_6_-benzylaminopurin and 0.25 mg/L α-naphthalene acetic acid for highest shoot regeneration (100% cultures responded with a mean number of 26.5 shoots per explant). Maximum rooting frequency (82%) and number (20.8) were obtained on half-strength MS medium with 1.0 mg/L indole-3-butyric acid. The rooted plants were acclimatized and transferred to the field. The HPTLC and SCoT analysis revealed the phytochemical and clonal similarity between the in vitro propagated plants and mother plant.

**Conclusions:**

In this study, it is confirmed that cotyledon is an excellent explant for direct and indirect shoot organogenesis in *T. siliquosa*. For direct shoot induction WPM and indirect organogenesis, MS medium was found to give better response. The true-to-type nature of in vitro-derived plants were confirmed by phytochemical and SCoT analysis. The protocol described here could be used for the large-scale propagation of elite clones of *T. siliquosa*.

**Supplementary Information:**

The online version contains supplementary material available at 10.1186/s43141-023-00523-8.

## Background

*Thottea siliquosa* (Lamk.) Ding Hou, belonging to the family Aristolochiaceae, is a slender erect shrub growing in the forests of Indian subcontinent, particularly in the Western Ghats of India and Sri Lanka. The genus *Thottea* has about 35 species, and in India, it is represented by about 14 species of which 10 are endemic to peninsular India [1]. *T. siliquosa* is widely used by different tribal communities in various parts of India to cure several ailments and is an important medicinal plant in the Ayurvedic system of medicine [2]. The root paste of *T. siliquosa* is applied externally for headaches and against spider, scorpion and snake poison. The root decoction is taken internally for chest pain and cough [3]. Powdered root in warm water is administered orally as an antidote to poisonous bites [4]. In Ayurveda, the roots of *T. siliquosa* are used against cholera, diarrhoea and dysentery, and the ointment prepared using the plant extracts is helpful for carbuncles and chronic ulcers [5].

Recently, the interest in phytochemical and pharmacological research of this species has increased. The antimicrobial, antifungal and antioxidant activities have been well documented in this plant [6]. The nonpolar solvent extracts of *T. siliquosa* showed cytotoxic properties [7]. Furthermore, the leaves and root extracts of *T. siliquosa* exhibited notable antioxidant and free-radical scavenging activities [8]. The root extracts of *T. siliquosa* showed anticancer and antioxidant properties [9]*.* Antigenotoxic and anti-inflammatory properties of methanolic extract of *T. siliquosa* have been reported [10]*.*

There are several limitations to the traditional propagation of this species. The destructive harvesting of *T. siliquosa* for its roots coupled with low seed germination rate, seedling survival and habitat destruction is the direct threat to the survival of this plant [11]. Since vegetative propagation through stem cutting is not successful, the rootstock is commonly used for the natural propagation of different species of *Thottea* including *T. siliquosa*. However, root stock is susceptible to pathogens and pests, and the success is low [12]*.* The collection of root stock often adversely affects the survival of *T. siliquosa*. This plant requires special habitats such as evergreen to semievergreen forests and stream sides for its growth and propagation; thus, it shows less regeneration potential in natural conditions, i.e. 11.0 regenerating seedlings per hectare in the forest [11].

Micropropagation techniques can be used for mass propagation, genetic improvement, disease-free plant production and conservation of various plant taxa [13]. This method also enables seasonless production and analysis of plant materials for their pharmaceutical applications. The species, explants and endogenous hormones influence the response of plant tissues, organs or cells towards in vitro propagation efforts [14]. Since every system is unique, the media, concentrations and combinations of plant growth regulators (PGRs) should be standardized for the successful in vitro propagation of individual species. In micropropagation, the photosynthetic ability of regenerated plants is influenced by the carbon source, PGRs and acclimatization conditions. For the optimization of micropropagation conditions, the evaluation of photosynthetic pigments is important [15].

Genetic and phytochemical stability assessment is essential to confirm the true-to-type nature of plants during in vitro mass propagation as it may vary during the culture process. The phytochemical stability studies using thin-layer chromatography (TLC) and high-performance thin-layer chromatography (HPTLC) profiling ensure the medicinal and nutritional qualities of daughter plants [16]. Molecular markers like restriction fragment length polymorphism (RFLP), inter-simple sequence repeats (ISSR), random amplified polymorphic DNA (RAPD) and start codon-targeted marker (SCoT)-based analysis can further confirm the genetic fidelity of regenerated plants [17].

The root of this plant is used for various medicinal purposes, and the plant has to be destroyed for collecting its roots. The indiscriminate collection often hampers its survival in the wild, and there is a pressure on the existing population of *T. siliquosa*. Therefore, micropropagation is regarded as a viable option for the rapid propagation of this plant. To our knowledge, there is no report on the micropropagation of *T. siliquosa*. Therefore, we have standardized protocols for the efficient in vitro propagation of *T. siliquosa* via. direct and indirect organogenesis using cotyledon explants.

## Methods

### Plant material and sterilization

Mature fruits were selected and harvested from the plants growing in Ranipuram hills, Kasaragod district, Kerala, India (latitude 12°41′ N and longitude 75°36′ E) from November to February. The species was identified, and the voucher specimen (no. CU 7090) has been submitted to Calicut University Herbarium, Kerala, India. The fruits were sun-dried for 3 days. The seeds were collected from dried fruits and were surface sterilized using 2.0% sodium hypochlorite (NaOCl, HiMedia, India) for 15 min followed by 0.1% mercuric chloride (HgCl_2_, HiMedia, India) solution for 5 min. The seeds were rinsed three times in sterilized distilled water and inoculated on Murashige and Skoog basal medium (MS, cat. no. PT099, HiMedia, India; [18]) in a sterile disposable petri dish (90 mm, Tarsons). About 25-mL media were poured into a petri dish, and 12 seeds were placed on one petri dish after sterilization. The seeds were kept for germination in the same growth conditions used for micropropagation. Seeds started to germinate 14 days after inoculation. About 40% of seeds germinated in the given medium and conditions. From the seedlings, cotyledons were excised and used for inoculation.

### Exudation control

The browning of media due to phenolic exudation, which leads to the death of explants, was observed during the culture experiments. To reduce the exudation effect, different concentrations of activated charcoal (*AC*, 5.0–15 mg/L), antioxidants such as polyvinylpyrrolidone (*PVP*, 100–300 mg/L) and ascorbic acid (20–60 mg/L; HiMedia, India) were added to the media during direct and indirect shoot regeneration (Table 1). Medium without any antioxidants and AC served as control. The observations were recorded after 6 weeks.

**Table 1:** Effect of various treatments to alleviate phenolic exudation during cotyledon culture for direct and indirect shoot induction

Treatment	Concentration (mg/L)	^a^Extent of exudation		**Survival rate (%)	
		Direct	Indirect	Direct	Indirect
Control	0.0	+ + + +	+ + + + +	16 g	6i
Ascorbic acid	20	+	+ + +	70b	65c
Ascorbic acid	40	-	+	92a	93a
Ascorbic acid	60	+	+ +	60c	72b
PVP	100	+ +	+ + +	26f	20 h
PVP	200	+ +	+ +	38e	30f
PVP	300	+ +	+ + +	36e	28 fg
Activated charcoal	5	+ + +	+ + + +	68b	57d
Activated charcoal	10	+	+ + +	47d	41e
Activated charcoal	15	+ +	+ + +	24f	26 g

For direct shoot induction WPM and indirect shoot induction, MS media were used

^a^The increase in “ + ” sign shows a corresponding increase in exudation. The sign “-” indicates no exudation

^**^The values are represented as the means ± SE of three independent experiments. Means followed by the same letters within the column are not significantly different (*p* ≤ 0.05) according to Duncan’s multiple range test

### Direct shoot induction

MS or Woody plant medium (WPM, cat. no. PT105, HiMedia, India; [19]) supplemented with various concentrations (0.5–2.0 mg/L) of N_6_-benzylaminopurin (BA), kinetin (KN) and thidiazuron (TDZ) alone or in combination with α-naphthalene acetic acid (0.1–0.5 mg/L, NAA) was used for direct shoot induction from cotyledon (Table 2). The shoot induction percentage, number of shoots and mean shoot length were recorded after 6 weeks. The explants with tiny shoots were subcultured on WPM supplemented with 0.4 mg/L TDZ for shoot elongation after 6 weeks in a glass jar (6.0 cm × 10.0 cm). Single explant was placed in the jar containing 70.0-mL medium. The number of shoots and mean shoot length were evaluated after 4 weeks of subculture. To assess the influence of cotyledon age on direct shoot regeneration, 3-, 5-, 7-, 9- and 11-day-old cotyledons were cultured on WPM supplemented with 1.0 mg/L TDZ and 0.25 mg/L NAA and evaluated the response after 6 weeks.

**Table 2:** Effect of different concentrations and combinations of PGRs on direct shoot induction from cotyledon explants of *T. siliquosa* in WPM and MS media after 6 weeks of culture with 40 mg/L ascorbic acid

Concentration of PGRs (mg/L)				*Percentage of response		*Number of shoots per explant		*Shoot length (cm)	
BA	KN	TDZ	NAA	WPM	MS	WPM	MS	WPM	MS
0.0	0.0	0.0	0.0	0.00	0.0	0.00	0.00	0.00	0.00
0.5				47f	38ef	3.7 ± 0.8d	2.0 ± 0.50ef	0.6 ± 1.24bc	1.0 ± 0.12b
1.0				58de	46e	6.2 ± 4.5bc	6.2 ± 0.4 cd	1.0 ± 0.56b	0.8 ± 0.22c
1.5				52e	22gh	5.3 ± 3.21 cd	4.5 ± 0.53d	0.7 ± 0.23bc	1.2 ± 0.1ab
2.0				17i	9ij	4.1 ± 14 cd	3.2 ± 0.23e	0.4 ± 0.43c	0.6 ± 0.04 cd
	0.5			8j	3j	2.8 ± 0.23de	1.3 ± 0.46f	0.3 ± 0.28 cd	0.68 ± 0.23 cd
	1.0			12i	6ij	2.0 ± 0.12e	2.0 ± 0.54ef	0.4 ± 0.12c	0.56 ± 0.56d
	1.5			23 h	20gh	2.8 ± 0.17de	2.3 ± 0.40ef	0.2 ± 0.27 cd	1.1 ± 0.32b
	2.0			20hi	12i	1.6 ± 0.14ef	1.2 ± 0.26f	1.1 ± 0.3b	0.7 ± 0.08 cd
		0.5		56de	42ef	10.8 ± 4.42b	4.8 ± 0.23d	0.5 ± 0.32c	0.9 ± 0.03bc
		1.0		72 cd	62c	12.6 ± 3.78ab	6.3 ± 0.34 cd	1.3 ± 0.32ab	1.1 ± 0.09b
		1.5		64d	56d	8.5 ± 0.76bc	5.9 ± 0.38 cd	1.4 ± 0.19ab	0.9 ± 0.05bc
		2.0		37 g	32f	4.0 ± 1.1d	2.0 ± 0.50ef	0.9 ± 0.48bc	1.0 ± 0.01b
1.0			0.1	75c	42ef	7.5 ± 0.18bc	9.2 ± 0.54c	1.0 ± 0.12b	0.9 ± 0.42bc
1.0			0.25	77bc	48e	8.7 ± 0.31bc	10.4 ± 0.67b	1.1 ± 0.72b	1.0 ± 0.96b
1.0			0.50	73 cd	34f	5.9 ± 0.63c	8.3 ± 0.34 cd	1.2 ± 0.42ab	1.2 ± 0.87ab
	1.5		0.1	25 h	23 g	2.8 ± 0.03de	4.2 ± 0.32de	0.3 ± 0.23 cd	0.3 ± 0.34de
	1.5		0.25	30gh	25 g	3.2 ± 0.14de	3.8 ± 0.12de	0.6 ± 0.67bc	0.5 ± 0.08d
	1.5		0.50	28 h	20gh	4.7 ± 0.67 cd	2.8 ± 0.09ef	0.9 ± 0.25bc	0.8 ± 0.02bc
		1.0	0.1	86b	72b	16.4 ± 0.41ab	10.3 ± 0.13b	1.3 ± 0.63ab	1.1 ± 0.47ab
		1.0	0.25	92a	78a	20.9 ± 0.32a	12.9 ± 0.56a	1.6 ± 0.77a	1.4 ± 076a
		1.0	0.50	84b	75b	16.4 ± 0.85b	10.6 ± 0.38b	1.6 ± 0.32a	1.2 ± 0.65ab

*MS*, medium; *WP*, WPM. ^*^The values are represented as the means ± SE of three independent experiments. Mean values within a column followed by the same letter are not significantly different (*p* ≤ 0.05) according to Duncan’s multiple range test

### Indirect shoot regeneration

Cotyledons at an age of 7 days were cultured in petri plates on MS medium fortified with various concentrations (0.25–0.75 mg/L) of BA or KN in combination with 2,4-dichlorophenoxy acetic acid (2,4-D, 0.5–1.5 mg/L) or NAA (0.5–1.5 mg/L) for callus induction (Table 3). The frequency of callus induction, fresh weight, dry weight and nature of calli was assessed after 5 weeks. Shoot initiation started after 5 weeks in the same medium, and number of shoot buds initiated was evaluated after 7 weeks. The calli with minute shoots were transferred to glass jar on MS medium augmented with BA or TDZ (0.25–1.5 mg/L) alone or in combination with NAA (0.1–1.0 mg/L) after 7 weeks for further growth and development of the shoots. The number of shoots and shoot length were recorded after 6 weeks of subculture.

**Table 3:** Effect of various PGRs on callus induction and shoot regeneration on MS medium with 40 mg/L ascorbic acid

Concentration of PGRs (mg/L)				*Percentage of response	*Fresh weight (mg)	*Dry weight (mg)	Shoot buds initiated per explant after 7 weeks of culture	
BA	KN	NAA	2,4-D				*Percentage of response	*Number of shoots
0.0	0.00	0.00	0.00	0.00	0.00	0.00	0.00	0.00
0.25		0.5		9 g	623 ± 1.68i	64 ± 0.9f	0.00	0.00
0.5		1.0		18ef	782 ± 1.9f	78 ± 0.7d	0.00	0.00
0.75		1.5		27e	846 ± 0.8c	83 ± 0.2 cd	67c	4.0 ± 0.43c
0.25			0.5	54c	924 ± 0.5b	90 ± 0.2ab	69d	9.6 ± 0.28b
0.5			1.0	81a	948 ± 0.8a	93 ± 0.5a	84a	12.8 ± 0.87a
0.75			1.5	66b	928 ± 0.6b	89 ± 0.4b	81b	6.1 ± 0.42bc
	0.25	0.5		16f	765 ± 0.6 h	72 ± 0.9ef	0.00	0.00
	0.5	1.0		36d	790 ± 1.7ef	77 ± 0.5de	0.00	0.00
	0.75	1.5		22ef	774 ± 1.3 g	73 ± 0.2e	0.00	0.00
	0.25		0.5	25e	795 ± 0.7e	75 ± 0.3de	29e	2.3 ± 0.48d
	0.5		1.0	56c	816 ± 0.9d	81 ± 0.4c	34de	1.8 ± 0.32d
	0.75		1.5	34d	802 ± 0.8de	83 ± 0.8 cd	47d	1.2 ± 0.41e

^*^The values are represented as the means ± SE of three independent experiments. Means followed by the same letters within the column are not significantly different (*p* ≤ 0.05) according to Duncan’s multiple range test

### Rooting and acclimatization

The shoots above 4.0 cm in length derived from direct and indirect organogenesis were cultured on half-strength MS medium supplemented with different concentrations (0.25–2.0 mg/L) of NAA or indole-3-butyric acid (IBA, Merck, India) for rooting (Table 4). The percentage of root induction, mean number of roots per shoot and root length were evaluated after 6 weeks. The well-rooted plantlets were taken out from the culture tubes and washed thoroughly to remove the adhering medium completely. Subsequently, they were transferred to paper cups (8.0 cm × 7.0 cm) containing autoclaved garden soil:organic manure:cocopeat in the ratio 1:1:1, covered in polythene bags with holes for maintaining humidity and kept inside the culture room at 22 ± 2 °C temperature under a 16/8-h (light/dark cycle) photoperiod using white, cool fluorescent lamps (50 μmol m^−2^ s^−1^ photon flux density) and 40–50% relative humidity. The plants were watered every alternate day with approximately 10.0 mL of distilled water and removed the polythene cover after 4 weeks. The plants were transferred to larger pots in a greenhouse at room temperature (25 °C) after 8 weeks and eventually transferred to the field after 6 months under shade condition.

**Table 4:** The effect of various concentrations of IBA and NAA in rooting of shoots on half-strength MS medium after 6 weeks

Auxin	Concentration (mg/L)	*Percentage of response	*Number of roots per shoot	*Root length (cm)	^a^Extent of callusing
IBA	0.00	0.00	0.00	0.0	
	0.25	22c	4.1 ± 0.34 cd	3.0 ± 0.34bc	-
	0.5	63b	12.2 ± 0.5b	3.4 ± 0.48b	-
	1.0	82a	20.8 ± 0.56a	4.8 ± 0.83a	-
	1.5	60b	6.2 ± 0.32c	2.6 ± 0.34c	+
	2.0	15de	2.16 ± 0.2de	2.46 ± 0.20 cd	+ + +
NAA	0.25	10e	1.0 ± 0.24e	2.2 ± 0.34 cd	-
	0.5	20c	1.5 ± 0.08de	3.1 ± 0.73bc	-
	1.0	18 cd	2.3 ± 0.1d	3.2 ± 0.32b	+
	1.5	16d	2.1 ± 0.23d	2.7 ± 0.19c	+ + +
	2.0	12ef	1.6 ± 0.16de	1.2 ± 0.13d	+ + + +

^*^The values are represented as the means ± SE of three independent experiments. Means followed by the same letter within the column are not significantly different (*p* ≤ 0.05) according to Duncan’s multiple range test

^a^The increase in “ + ” sign shows a corresponding increase in callus induction

### Histological analysis

Cotyledon containing direct shoots and calli with shoots were fixed in formalin, acetic acid and alcohol mixture (*FAA*, 90.0-mL 70% ethanol, 5.0-mL acetic acid and 5.0-mL formalin). Freehand sections were taken from fixed samples, stained with safranin and observed under trinocular light microscope (Olympus, Japan). The pictures were captured using Magnus camera (Magnus, India).

### Estimation of chlorophyll content

The chlorophyll content in the mother plant and in vitro raised plants was estimated according to the procedure of Arnon [20]. About 500 mg of fresh leaf tissues was collected from the mother plant and 3-month-old in vitro-derived plants. The leaf tissues were homogenized in 10.0 mL of chilled 80% (*v/v*) acetone using a motor and pestle. About 50.0 mg of calcium carbonate (CaCO_3_: HiMedia, India) was added during the grinding. The extract was collected and centrifuged at 4000 rpm for 5 min in room temperature. The supernatant was collected, and the pellet was resuspended in 5.0 mL of the 80% acetone and extracted thrice. The supernatants were pooled and made up to 25.0 mL using 80% acetone in a volumetric flask. The absorbance was measured at 645 and 663 nm using a spectrophotometer (Shimadzu UV 2600, Japan). Acetone (80%) was placed as control.

The amount of chlorophyll a, chlorophyll b and total chlorophyll was calculated using the equation as follows:

$$\begin{array}{*{20}c} {{\text{Chlorophyll a}}:{ 12}.{7 }\left( {{\text{A663}}} \right){-}{2}.{69 }\left( {{\text{A645}}} \right) \, \times {\text{V}}/{1}000 \times {\text{W}}} \\ {{\text{Chlorophyll b}}:{ 22}.{9 }\left( {{\text{A645}}} \right){-}{4}.{68 }\left( {{\text{A663}}} \right) \, \times {\text{V}}/{1}000 \times {\text{W}}} \\ {{\text{Total chlorophyll}}:{ 2}0.{2 }\left( {{\text{A645}}} \right) \, + {8}.0{2 }\left( {{\text{A663}}} \right) \, \times {\text{V}}/{1}000 \times {\text{W}}} \\ \end{array}$$

where A: absorbance, V: volume of sample in mL and W: weight of sample in g.

### HPTLC fingerprinting

The roots of mother plant and randomly selected in vitro generated plants (both direct and indirect) were collected, washed and shade dried. The extracts were prepared by grinding the samples in methanol. The extracts were filtered and dried in hot air oven at 50 °C. The dried extracts were weighed and dissolved in methanol (1.0 mg/mL) for further experiments. The aristolochic acid 1 (200 µg/mL; Sigma, cat. no. A5512, USA) was used as marker compound. Silica-based TLC 60-F 254 plates (200 × 100 mm; Merck, India) activated by heating at 110 °C for 15 min were used for separation. Samples were applied on TLC plates in bands using 10-µl syringe (Camag Linomat 5, Switzerland). The development of the chromatogram was carried out according to Agrawal and Laddha [21]. The mobile phase consists of *n*-hexane:chloroform:methanol (1:8:1 *v/v*). The twin trough chamber was saturated with 20.0 mL of solvent system for 20 min at room temperature (25 °C) and 40% relative humidity. After development, plates were dried and visualized in both 254 nm and 366 nm without derivatization. Furthermore, the plates were visualized in visible light and 366 nm after derivatization using anisaldehyde sulphuric acid reagent in photo-documentation chamber (Camag Scanner 4 and visualizer 1, Switzerland).

### SCoT analysis

SCoT molecular marker analysis was carried out to confirm the genetic fidelity of in vitro regenerated plants. Plants regenerated through direct and indirect methods were randomly selected and compared with the mother plant. The cetyltrimethylammonium bromide (CTAB; [22]) method with slight modification by adding 2% polyvinylpyrrolidone (PVP; Merck, India) was used for the extraction of total genomic DNA from the fresh leaves. The concentration of DNA was estimated by measuring the absorbance at 260 nm, and the purity of DNA was determined by finding the ratio of absorbance at 260 and 280 nm using a NanoDrop (Thermo Fisher Scientific, USA). DNA samples were prepared to a concentration of 50–100 ng/μL and stored at ^−^20 °C for further analysis. The PCR amplification of 1–20 SCoT primers [Integrated DNA Technologies (IDT)] was carried out in the thermal cycler (ProFlex PCR system, Thermo fisher Scientific, USA) in 20.0-µL reaction mixture that contains 2.0 µL 10 × PCR buffer, 1.6-µL primer (10.0 µM), 1.6-µL dNTPs (10 mM), 0.3-µL Taq DNA polymerase (3 units/µL), 3.0-µL template DNA (50 ng/μL) and 11.5-µL molecular biology grade water (TaKaRa bio, India). The PCR conditions for SCoT analysis were the following: initial denaturation at 94 °C for 3 min followed by 34 cycles of 94 °C for 1 min, annealing temperature (*T*a °C) for 1 min, 72 °C for 2 min, final extension at 72 °C for 5 min and cool down to 4 °C. The *T*a was kept at 4 °C below the melting temperature (*T*m) of the particular primer sequence. The *T*m values of the primers ScoT 1 to ScoT 20 ranged from 52.3 to 60.8 °C. After amplification, 6.0-µL PCR products were mixed with 2.0 µL of 10 × gel loading dye (0.25% bromophenol blue, 0.25% xylene cyanol FF, 30% glycerol in water) and loaded on 1.2% agarose gel. The bands were separated in 1 × Tris/Borate/EDTA (TBE) buffer by electrophoresis. After electrophoresis, the gel was stained with ethidium bromide (0.5 µg/mL) for 20 min visualized in a gel documentation system (Bio-Rad, USA). The size of the amplicon was measured by comparing it with 1-kb DNA ladder (TaKaRa bio, India). The SCoT profiles were scored manually using binary matrix. Only clear and distinct bands were used for evaluation. With each primer, two independent amplification experiments were conducted.

### Culture conditions and statistical analysis

The culture medium pH was adjusted to 5.8 using 0.1-N NaOH or 0.1-N HCl, solidified with 8.0 g/L agar (HiMedia, India) and autoclaved at 121 °C and 1.06 kg/cm^2^ pressure for 20 min. The in vitro cultures were incubated in the laboratory at 22 ± 2 °C temperature under a 16/8-h (light/dark cycle) photoperiod using white, cool fluorescent lamps (50 μmol m^−2^ s^−1^ photon flux density) and 40–50% relative humidity. All the experiments were repeated thrice with a minimum of 20 explants per experiment, and PGR-free media were used as control. SPSS software version 24.0 (IBM, Chicago, IL, USA) was used for the statistical analyses. Data were analysed for the significance of differences of means among treatments using Duncan’s multiple range test (*DMRT*, [23]) at *P* ≤ 0.05. The results were presented as mean ± standard error (SE) of three replicates of the experiments.

## Results

### Seed germination and exudation control

**Fig. 1:**
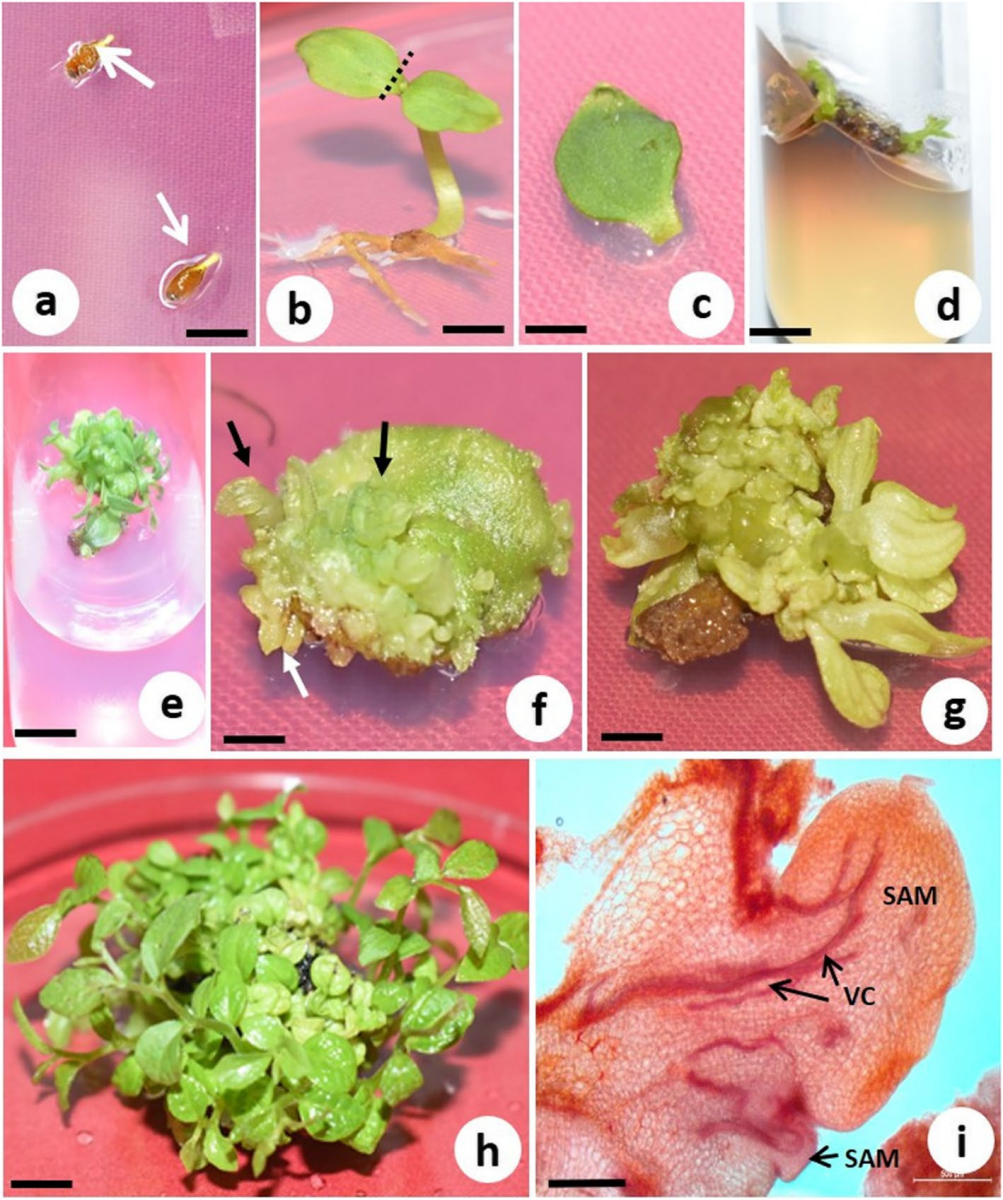
Direct shoot regeneration of *T. siliquosa* from cotyledon explants. **a** Germination of seeds (arrow) on MS basal medium after 1 week of culture (bar = 5 mm). **b** 7-day-old seedling with line of excision (dotted lines) of cotyledon (bar = 5 mm). **c** Cotyledon in the shoot induction medium consists of WPM with 1.0 mg/L TDZ and 0.25 mg/L NAA (bar = 2 mm). **d** Control culture (medium without ascorbic acid) showing browning due to phenolic exudation (bar = 8 mm). **e** Culture medium containing healthy shoots on optimum shoot induction medium with 40.0 mg/L ascorbic acid. There was no phenolic exudation (bar = 9 mm). **f** Shoot bud initiation (arrows) after 2 weeks of culture in the same medium as above. Shoot buds were originated from the explant (bar = 2.5 mm). **g** Same after 6 weeks of culture. Shoots elongated further (bar = 2.5 mm). **h** Subcultured shoots on WPM with 0.4 mg/L TDZ after 4 weeks. Several elongated shoots are visible (bar = 1 cm). **i** Histological analysis of direct shoot organogenesis showing multiple shoots with vascular connection (arrow) from the explant. SAM, shoot apical meristem; VC, vascular connection (bar = 500 µm)

Surface-sterilized seeds were inoculated on the hormone-free MS medium. The germination of seeds started 14 days after inoculation (Fig. 1a). The cotyledons of germinated seedlings were excised and cultured horizontally with its abaxial surface touching the medium in sterile disposable petri dishes (Fig. 1b, c). The exudation of phenolic compounds from the cut end of the cotyledons was a serious problem during cotyledon culture (Fig. 1d). To reduce exudation, various compounds like AC, PVP and ascorbic acid were used (Table 1). The browning of culture due to phenolic exudation adversely affected the survival and growth of cultures in direct and indirect shoot regeneration. The addition of PVP (100–300 mg/L) in the medium showed better exudation control but lower survival of cultures than media with AC (5–15 mg/L). Among the different chemicals at various concentrations tested, 40.0 mg/L ascorbic acid was found optimum with no exudation in the culture and highest survival rate of explants in both direct (92%) and indirect (93%) shoot induction (Fig. 1e, Table 1). Hence, all further culture initiation experiments with cotyledon were done with medium containing 40.0 mg/L ascorbic acid.

### Seedling age and direct shoot regeneration

**Fig. 2:**
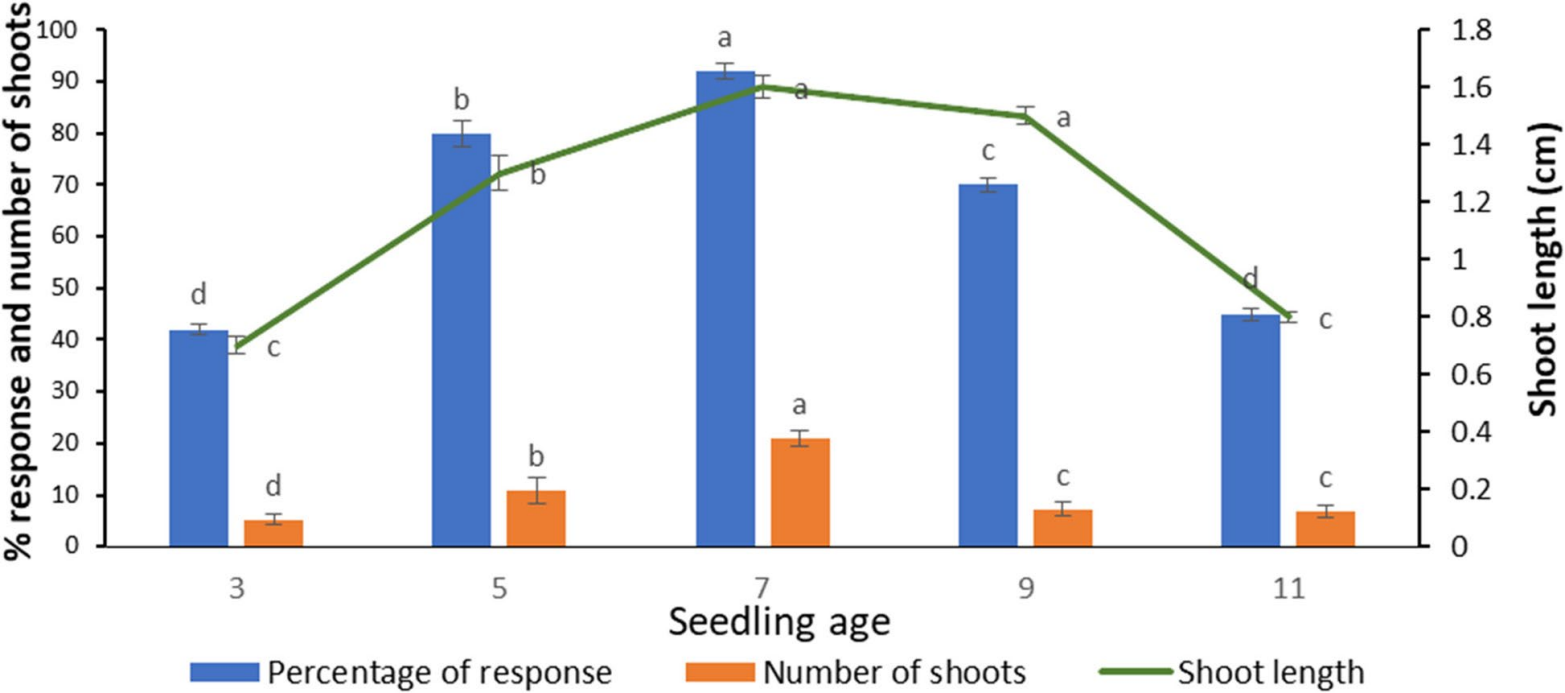
Effect of age of seedling on direct shoot induction from cotyledon explants of *T. siliquosa* 6 weeks after culture on WPM supplemented with 1.0 mg/L TDZ and 0.25 mg/L NAA. The data in the bar graphs are presented as means ± SE of three independent experiments. Different letters indicate significant differences, as determined using Duncan’s multiple range test (*P* ≤ 0.05)

The cotyledons from 3-, 5-, 7-, 9- and 11-day-old seedlings were inoculated on WPM supplemented with 1.0 mg/L TDZ and 0.25 mg/L NAA. The results showed that the seedling age greatly influenced the direct shoot induction frequency and the number of shoots formed from the cotyledon. The highest shoot induction was obtained by using 7-day-old seedlings with 92% induction frequency, 20.9 mean shoot number per explant and an average shoot length of 1.6 cm after 6 weeks of culture (Fig. 2). The cotyledons collected before and after 7 days gave significantly lower shoot induction frequency and number of shoots. Since the optimum shoot induction was obtained from cotyledons collected from 7-day-old seedlings, all further experiments were conducted with such cotyledons.

### Effect of media and PGRs in the direct shoot regeneration

For the direct shoot induction, WPM and MS medium supplemented with various concentrations and combinations of BA, KN, TDZ (0.5–2.0 mg/L), NAA (0.1–0.5 mg/L) and 40.0 mg/L ascorbic acid were used. On PGR-free medium, there was no shoot induction. Individual use of BA, KN and TDZ produced shoots with low frequency and number. However, a combination of auxin and cytokinin significantly improved the shooting performance of the cotyledon (Table 2). Of the various combinations of auxin and cytokinin tested, 1.0 mg/L TDZ and 0.25 mg/L NAA in WPM produced the highest response. On this medium, 92% of explants responded with a mean number of 20.9 of shoots per explant within 6 weeks of culture (Table 2, Fig. 1f, g). Comparatively, WPM was better than MS medium in terms of percent response and the number of shoots. Callus formation was observed in the MS medium at higher concentrations of PGRs. Although shoot induction was observed on all media except basal media, shoot elongation was poor. Therefore, these cultures were transferred to WPM supplemented with 0.4 mg/L TDZ for shoot elongation. On this medium, 27.6 shoots were produced with a mean shoot length of 4.8 cm after 4 weeks of transfer (Fig. 1h).

The histological analysis revealed the emergence of shoot apical meristem (SAM) from the cotyledon explants. It also showed vascular connection (VC) between the explant and the SAM (Fig. 1i).

### Callus-mediated shoot regeneration

**Fig. 3:**
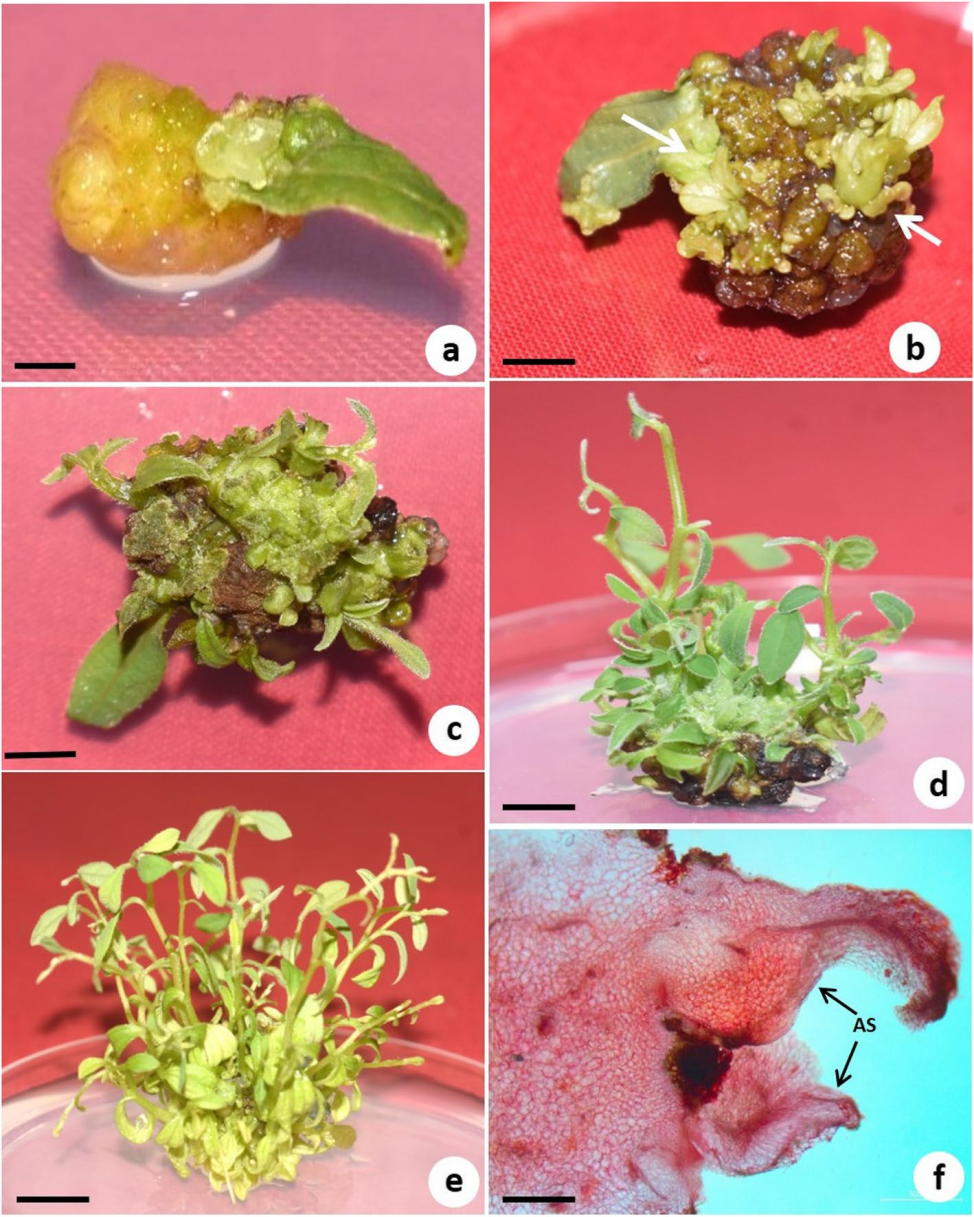
Callus-mediated shoot regeneration from cotyledon explants of *T. siliquosa*. **a** Callus formed at the cut end of cotyledon on MS medium supplemented with 1.0 mg/L 2,4-D and 0.5 mg/L BA after 2 weeks (bar = 2 mm). **b** Seven-week-old green/brown, compact and nodular callus with minute shoot buds (arrow) (bar = 2 mm). **c** Differentiating callus on MS medium containing 0.5 mg/L BA and 0.25 mg/L NAA for shoot proliferation after 2 weeks (bar = 3 mm). **d** Same culture after 4 weeks. Shoots have elongated (bar = 5 mm). **e** Large number of well-developed multiple shoots differentiated from callus after 6 weeks (bar = 1 cm). **f** Histology of callus showing regenerated shoot bud (bar = 500 µm)

To induce callus from 7-day-old cotyledons, MS medium supplemented with various concentrations (0.25–0.75 mg/L) of BA or KN in combination with 2,4-D or NAA (0.5–1.5 mg/L) and 40.0 mg/L ascorbic acid was tested. Both callus induction and shoot initiation occurred on the same medium. Among the various concentrations and combinations of PGRs tested, MS medium augmented with 1.0 mg/L 2,4-D, and 0.5 mg/L BA was found most effective in inducing green–brown, nodular and compact organogenic calli (81%) with shoots (84% shoot induction with 12.8 shoots per explant). Callus with the highest fresh weight (948 mg) and dry weight (93 mg) was also obtained on this medium (Table 3). Callus was induced from the cut end of the explant in all experiments except MS basal medium (control) after 1 week of culture (Fig. 3a). Shoot buds started forming after 5 weeks of culture in the same medium (Fig. 3b).

**Fig. 4:**
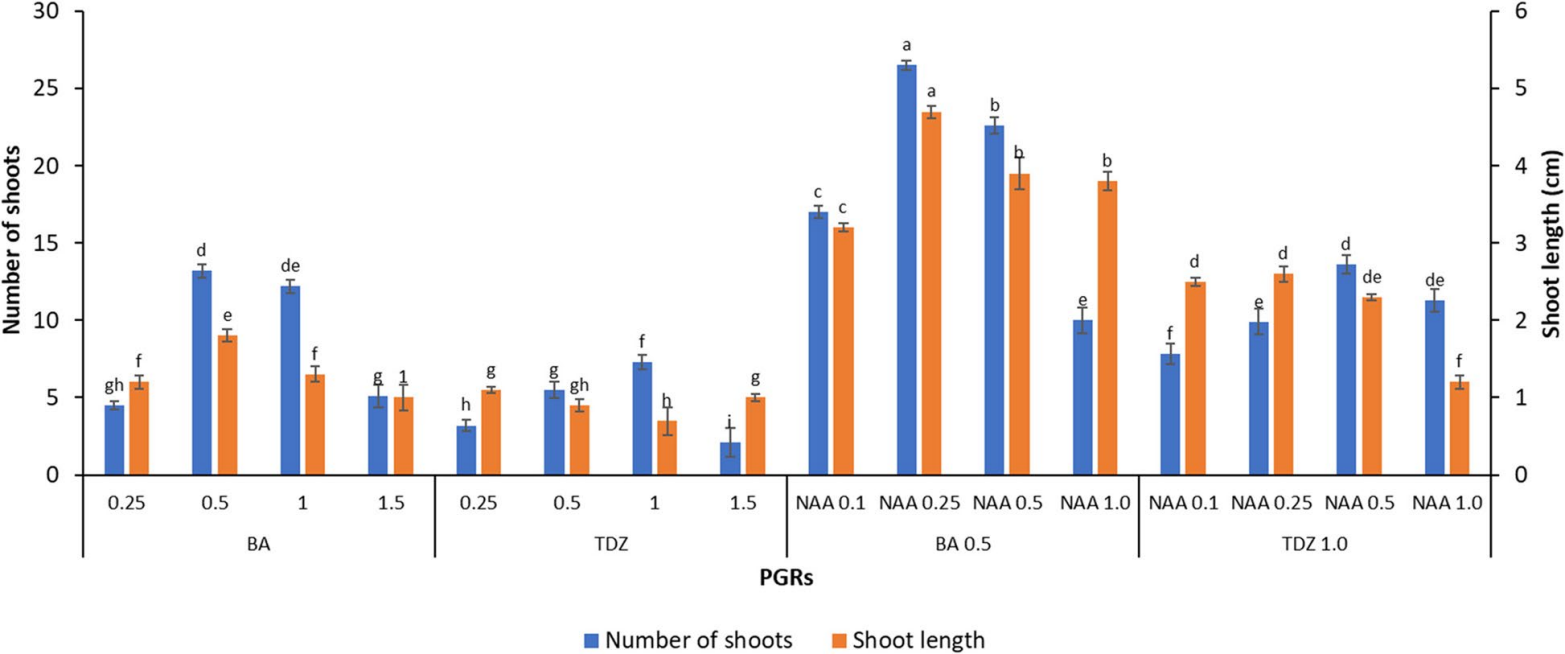
Effect of different combinations and concentrations of BA, TDZ and NAA on shoot bud proliferation and elongation from callus with minute shoots cultured on MS medium after 6 weeks of subculture. The data in the bar graphs are presented as means ± SE of three independent experiments. Different letters indicate significant differences, as determined using Duncan’s multiple range test (*P* ≤ 0.05)

The callus with minute shoots was transferred to shoot proliferation medium after 7 weeks. Among various concentrations (0.25–1.5 mg/L) and combinations of BA or TDZ with NAA (0.1–1.0 mg/L) on MS medium used for the subculture of callus with shoots, a combination of 0.5 mg/L BA and 0.25 mg/L NAA was found optimum for shoot multiplication and elongation (Fig. 3c, d). The highest shoot number, i.e. 26.5 and shoot length 4.7 cm, was observed on this medium (Fig. 3e; Fig. 4) after 6 weeks. The histological study revealed the induction of multiple shoots (MS) from the peripheral region of the callus, AS, Adventitious shoots (Fig. 3f).

### Rooting and acclimatization

**Fig. 5:**
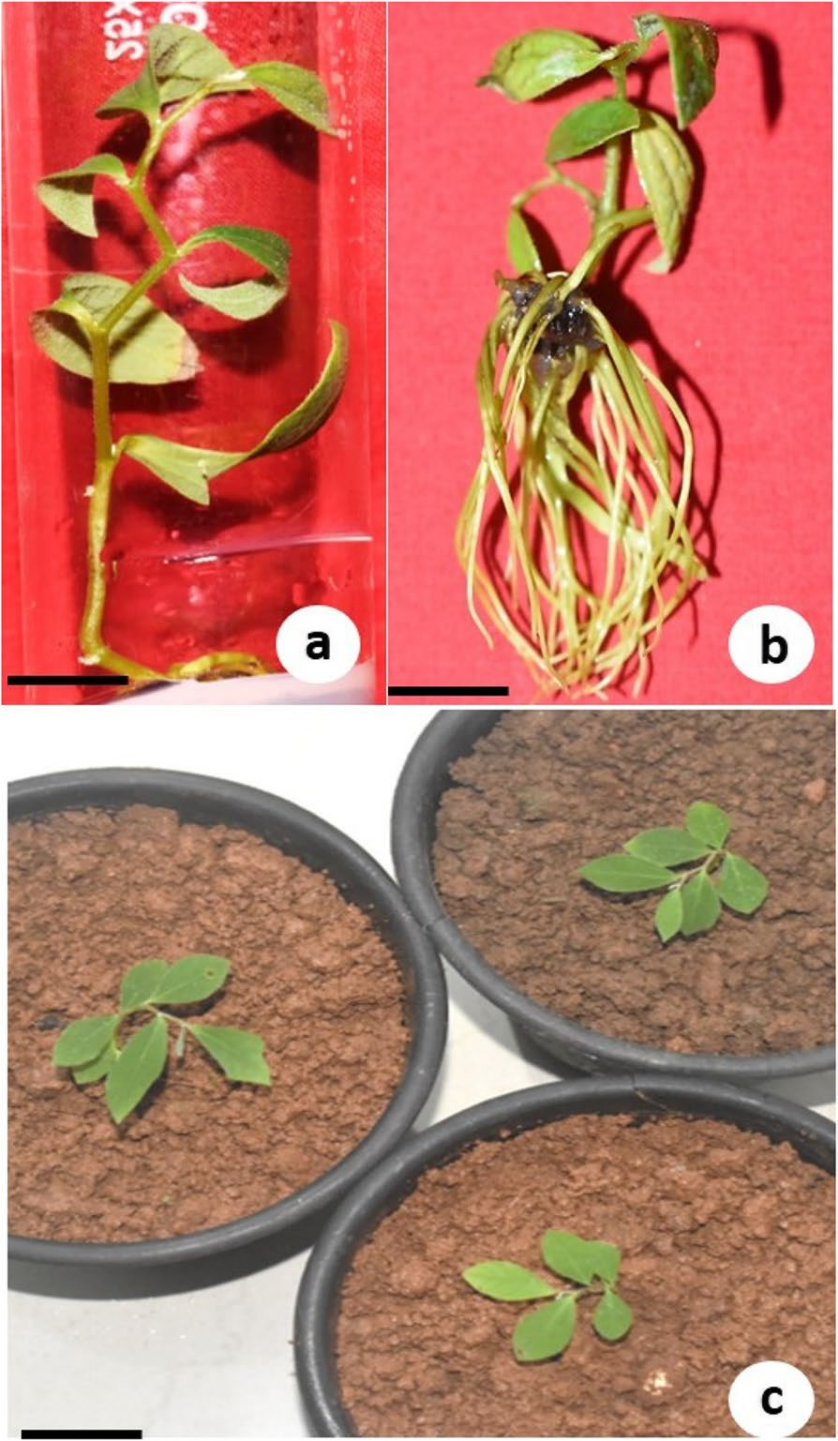
Rooting and acclimatization of in vitro propagated plants. **a** Isolated shoot on shoot elongation medium, i.e. WPM supplemented with 0.4 mg/L TDZ (bar = 1 cm). **b** Well-rooted shoot on half MS medium with 1.0 mg/L IBA after 6 weeks (bar = 1 cm). **c** Three acclimatized plants 2 months after transfer to soil (bar = 2.5 cm)

The in vitro regenerated shoots above 4.0 cm length were transferred to half-strength MS medium supplemented with various concentrations of (0.25–2.0 mg/L) IBA or NAA (Fig. 5a). The root initiation was observed after 2 weeks of culture. There was no root formation on basal medium (control). The highest rooting frequency (82%), mean number of roots per shoot (20.8) and root length (4.8 cm) were achieved on half-strength MS medium supplemented with 1.0 mg/L IBA after 6 weeks of culture (Table 4, Fig. 5b). IBA above 1.0 mg/L promoted callus formation from the basal cut end of the shoot. The rooted plantlets were established in the soil after acclimatization (Fig. 5c) with 87% survival rate. The shoots cultured on half-strength WPM failed to produce roots (data not shown).

### Chlorophyll content

**Fig. 6:**
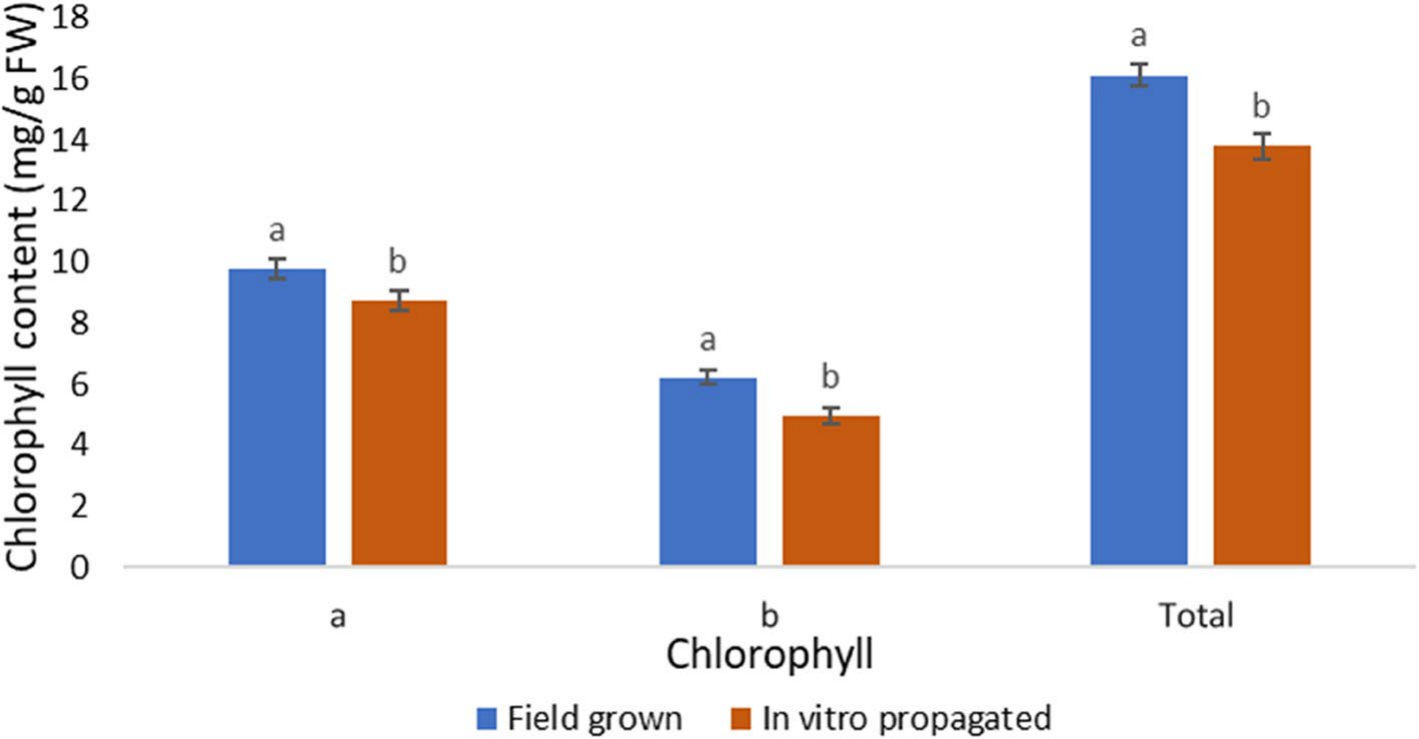
The chlorophyll content in field grown and in vitro propagated plants. The data in the bar graphs are presented as means ± SE of three independent experiments. Different letters indicate significant differences, as determined using Duncan’s multiple range test (*P* ≤ 0.05)

The total chlorophyll content of 3-month-old in vitro plants and mother plant was estimated. The chlorophyll a (9.78 mg/g), b (6.21 mg/g) and total chlorophyll (16.1 mg/g) were found highest in field grown plants compared to in vitro raised plants (Fig. 6). However, the chlorophyll a/b ratio was higher in micropropagated plants.

### HPTLC fingerprinting

**Fig. 7:**
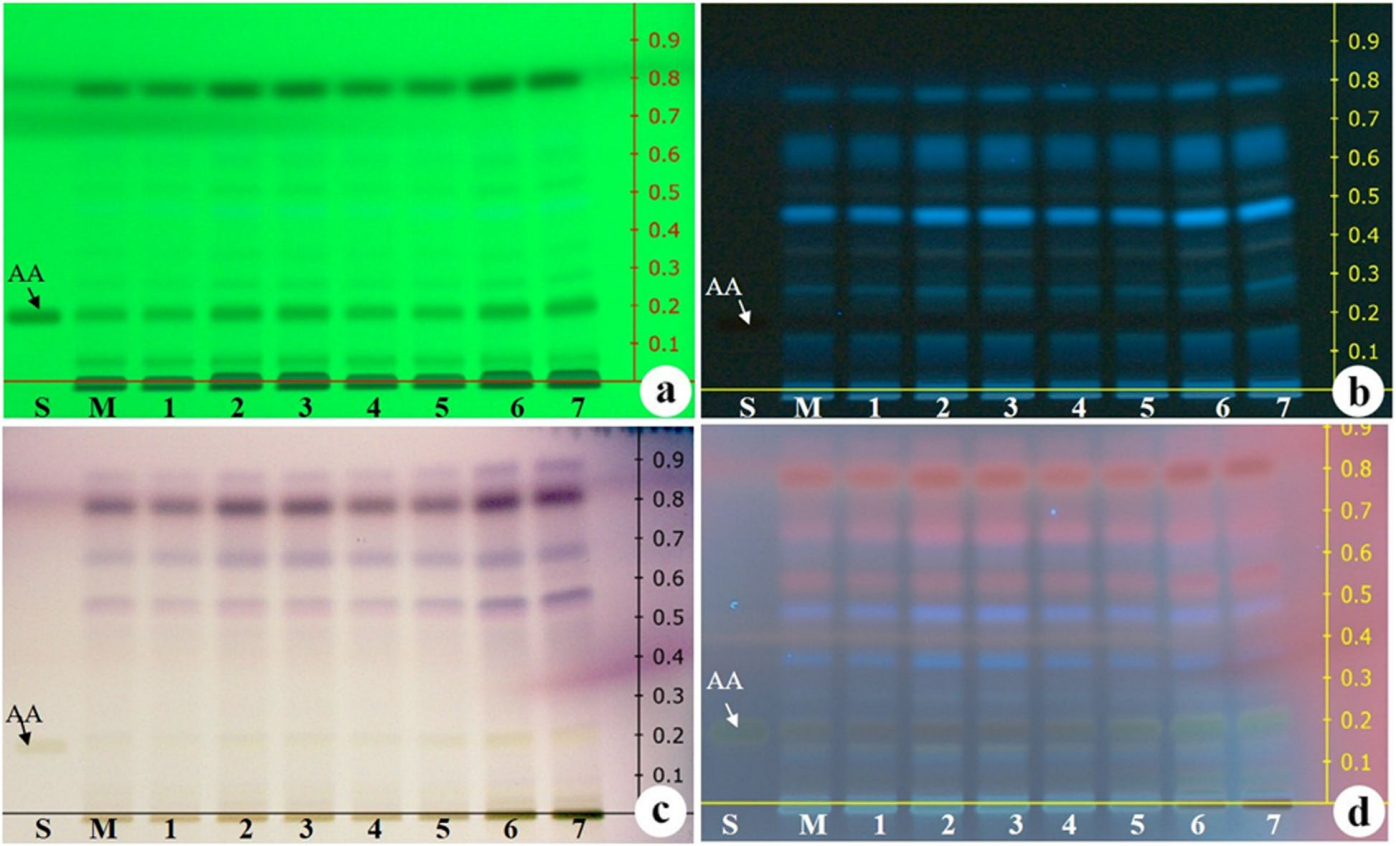
HPTLC chromatogram of methanolic root extracts. *S*, standard (*AA*, aristolochic acid 1); *M*, mother plant; *1–7 *in vitro propagated plants. **a** At 254 nm. **b** At 366 nm. **c** At visible light after derivatization. **d** At 366 nm after derivatization

The HPTLC fingerprinting of methanolic extracts of roots showed compact, well-separated bands of aristolochic acid 1 and other active phytochemicals at 254 nm and 366 nm (Fig. 7a, b) and in visible light and 366-nm derivatization with anisaldehyde sulphuric acid reagent (Fig. 7c, d) in the mobile phase *n*-hexane:chloroform:methanol (1:8:1 *v/v*). The fingerprint profile of in vitro propagated and mother plant showed similar band patterns. The presence of aristolochic acid 1 in both in vitro propagated and field-grown mother plant was confirmed by the corresponding bands in both plant extracts and standard at *R*_f_ value of 0.20.

### SCoT analysis

**Fig. 8:**
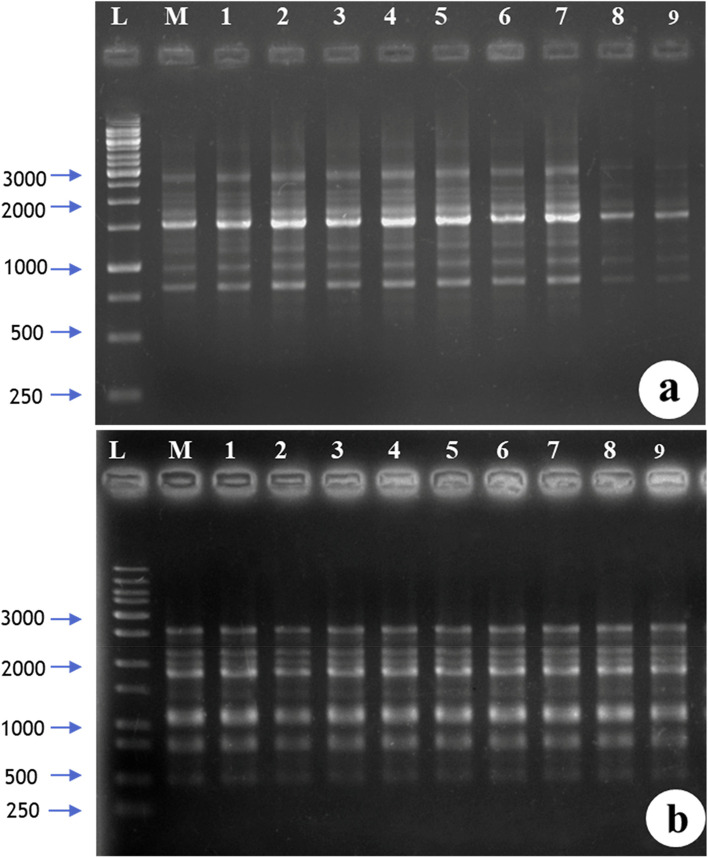
Representative SCoT amplification profile of field-grown mother plant and randomly selected in vitro grown plants. L, ladder; *M*, mother plants *1–9* in vitro grown plants. **a** SCoT 9. **b** SCoT 16

To confirm the genetic fidelity of in vitro regenerated plants, SCoT molecular marker-based profiling was carried out. Nine randomly selected plants and one mother plant were compared. Out of 20 primers tested, 14 produced reproducible bands with an average 3.57 bands per primer (Table 5). The band size ranges from 250 to 3000 bp. Polymorphic bands were completely absent. The mother plant and in vitro regenerated plants exhibited monomorphic band patterns, and therefore, no genetic variations were observed between these plants (Fig. 8a, b).

**Table 5:** List of primers used for SCoT analysis

Sl. no	Primer	Primer sequence (5′–3′)	Number of bands amplified	Approximate band length
1	SCoT 1	CAACAATGGCTACCACCA	3	1000–2500
2	SCoT 2	CAACAATGGCTACCACCC	3	600–2000
3	SCoT 4	CAACAATGGCTACCACCT	3	250–1500
4	SCoT 6	CAACAATGGCTACCACGC	3	600–1000
5	SCoT 7	CAACAATGGCTACCACGG	2	1200–2000
6	SCoT 8	CAACAATGGCTACCACGT	2	500–1500
7	SCoT 9	CAACAATGGCTACCAGCA	8	750–3000
8	SCoT 10	CAACAATGGCTACCAGCC	2	1400–2500
9	SCoT 11	AAGCAATGGCTACCACCA	2	750–3000
10	SCoT 12	ACGACATGGCGACCAACG	4	1000–2500
11	SCoT 13	ACGACATGGCGACCATCG	1	1000
12	SCoT 14	ACGACATGGCGACCACGC	4	500–2000
13	SCoT 15	ACGACATGGCGACCGCGA	6	250–2500
14	SCoT 16	ACCATGGCTACCACCGAC	7	500–2500

## Discussion

There is a considerable increase in the adoption of in vitro propagation techniques for the mass production and conservation of important medicinal plants over the past few decades [24]. Seed-derived explants like cotyledon, hypocotyl or cotyledonary node are often used for the in vitro propagation, conservation or genetic transformation of different plants [25]. The advantage of using cotyledon explants is the ease to get many sterile explants in a few days from germinating seedlings. Since the cotyledon is a part of the embryo, the cells of the cotyledons are young and active and have more vitality. It is considered the most responsive explant in various studies [26].

*T. siliquosa* contains high level of phenolic compounds [2]. The release of phenolic compounds through the cut ends of explants and callus oxidizes the tissue and causes media browning which significantly influences the results of in vitro culture [27]. Different methods can be adopted for the elimination of this phenomenon including media modification and continuous culture. In our study, the addition of ascorbic acid in media significantly controlled the phenolic exudation and resulted in higher explant survival followed by PVP. The medium without any antioxidants and AC (control) showed the highest browning and culture loss. Different studies reported the successful application of ascorbic acid to reduce the exudation of phenols and subsequent media and explant browning [28].

The age of the cotyledon plays a crucial role in shoot regeneration as the meristematic activity is higher in young cotyledon, and it decreases with an increase in age [29]. In the present study, the age of the cotyledon showed a significant effect on the rate of direct shoot induction and shoot number. Here, the optimum result was obtained on 7-day-old cotyledons. Similarly, the age of the cotyledon influenced the shoot regeneration ability in *Brassica rapa* [30], where the maximum results were obtained from 4-day-old cotyledons.

MS and WPM were tested for direct shoot induction, and the optimum result was obtained in WPM. WPM is specially developed for woody shrubs and trees which is characterized by having low salt concentration, and most woody plants are known to be sensitive to the total salt concentration of the media [31]. In the present study, the use of WPM reduced media browning compared to MS medium as in *Ilex guayusa* [32]. *T. siliquosa* is a woody shrub, and hence, it responded well in WPM than MS medium. WPM has been reported better than MS medium for multiple shoot induction in various species like *Vaccinium myrtillus* [33].

TDZ showed better regeneration potential than other cytokinins tested in this study. TDZ is a phenylurea-type synthetic PGR with cytokinin-like properties used in plant tissue culture [34]. TDZ is found to have superior or equally good shoot morphogenesis and callus dedifferentiation effect as compared to adenine-like cytokinins such as BA and KN in many species such as *Neolamarckia cadamba* [35] and *Phoenix dactylifera* [36]. In our study, the synergetic effect of TDZ and NAA enhanced shoot induction which agrees with the findings in different medicinal plants like *Capparis decidua* [37] and *Brassica rapa* [38]. The synergism might be attributed to the interaction between the hormones due to their differences in signaling pathways [38].

Fresh weight and dry weight are the two important parameters used for the growth measurement of callus cultures [39]. The results in the present study showed that the type and concentration of auxin (NAA and 2,4-D) have an important role in callus induction as well as the fresh and dry weight of the callus. This may be due to the undifferentiated tissue proliferation and its continued growth promoted by the presence of auxin [40]. A combination of 2,4-D and BA showed significant improvement in callus induction as well as shoot regeneration from callus. There are previous reports of the synergistic effect of 2,4-D and BA in the callus induction and shoot regeneration in other systems including *Larix olgensis* [41]. For the further shoot induction, proliferation and elongation, the MS medium augmented with BA in combination with NAA was found optimum which is in agreement with the results of Rajput et al. [42] who obtained shoot regeneration from cotyledon derived calli in *Sesamum indicum*.

In the present study, the optimum rooting of the shoots was obtained on half-strength MS medium with 1.0 mg/L IBA. Both above and below to the concentration reduced the rooting efficiency. IBA is an auxin commonly used to induce roots during micropropagation. In systems such as *Plumbago europaea* [43], IBA-induced rooting has been reported. Half-strength WPM caused defoliation of the shoots and loss of vigour. The shoots eventually died off. NAA produced callus from the basal cut end of the shoot at higher concentrations. Callusing at the time of rooting can cause a decrease in the adaptability of the plants and further developmental stages [44]. After successful acclimatization, the plants were transplanted to the field and observed normal growth.

The photosynthetic capacity of plants is greatly influenced by the chlorophyll content and its ratio. In micropropagated plants, the chlorophyll content can be considered as a tool for the evaluation of the physiological state of the plant as well as successful acclimatization [45]. In the present study, the amount of chlorophyll a, b and total chlorophyll content was lower than field-grown plants. However, the chlorophyll a/b ratio of in vitro grown plants was higher. The higher chlorophyll a/b ratio in micropropagated plants compared to mother plants showed the favourable condition during acclimatization which lead to optimum growth. Similar observations were made by Dilkalal in *Asystasia gangetica* [46].

The HPTLC fingerprint of plant extracts showed multiple bands with different colours and *R*_f_ values which confirmed the presence of various active phytochemical constituents in the methanolic root extract of mother plant and in vitro-derived plants. The HPTLC analysis confirmed the presence of aristolochic acid 1 in the in vitro propagated plants. Aristolochic acid is a bioactive phytochemical compound and an important biomarker present in the family Aristolochiaceae [47]. The similarity between the HPTLC fingerprints of mother plant and in vitro propagated plants showed the phytochemical stability between them. The similarity in HPTLC chromatogram of field grown and in vitro propagated plants were previously reported in several systems like *Dioscorea bulbifera* [48] and *Hedychium coronarium* [49].

Prolonged exposure to PGRs and long culture duration may cause DNA level variations in the in vitro grown plants, especially if the clonal plants were produced through indirect method [50]. Hence, PCR-based molecular marker studies are essential to confirm the genetic fidelity of regenerated plants to the mother plant. SCoT is a highly accurate, reliable and reproducible molecular marker compared to other routinely used molecular markers and are based on the start codons of the expressive sequence of the genome [51]. In the present study, the amplicons obtained from different primers gave monomorphic band patterns with the mother plants and randomly selected in vitro generated daughter plants. Thus, it showed genetic similarity between the mother plant and daughter plants. SCoT analysis is successfully employed for the genetic fidelity assessment during micropropagation studies in taxa like *Tylophora indica* [52] and *Prunus salicina* [53].

## Conclusions

The present study is the first report on the in vitro propagation of the important medicinal plant *T. siliquosa*. The cotyledon is an excellent explant to obtain direct and indirect regeneration in this plant. The advantage of using this protocol is to obtain large number of *T. siliquosa* plants through cotyledon-derived direct and indirect shoot induction. The exudation problem was minimized by using the antioxidant ascorbic acid. HPTLC fingerprinting technique was used to assess the phytochemical fidelity in *T. siliquosa*. Furthermore, the true-to-type nature of plants was confirmed by SCoT analysis. The application of this procedure can mitigate the pressure on the natural population and lead to further phytochemical, pharmacological and conservation studies of this species.

## Supplementary Information

**Additional file 1: Supplementary data.** SCoT 9: L: Ladder, M: Mother plant, 1–9: In vitro regenerated plants. SCoT 16: L: Ladder, M: Mother plant, 1–9: In vitro regenerated plants.

## Data Availability

The datasets generated during and/or analysed during the current study are available from the corresponding author on reasonable request.

## Abbreviations

2,4-D: 2,4-Dichlorophenoxyacetic acid
BA: N_6_-Benzylaminopurin
IBA: Indole-3-butyric acid
KN: Kinetin
MS: Murashige and Skoog medium
NAA: α-Naphthalene acetic acid
SE: Standard error
TDZ: Thidiazuron
WPM: Woody plant medium
SCoT: Start codon targeted
HPTLC: High-performance thin-layer chromatography
