# Supplementary material for: Control of media browning during micropropagation and assessment of biochemical and clonal fidelity of in vitro-derived and mother plants in Thottea siliquosa (Lamk.) Ding Hou., an important ethnomedicinal shrub
Source: J Genet Eng Biotechnol. 2023 Jun 2;21:70. doi: 10.1186/s43141-023-00523-8 (PMC10236102; doi:10.1186/s43141-023-00523-8)

SCoT 9: L: Ladder, M: Mother plant, 1-9: In vitro regenerated plants


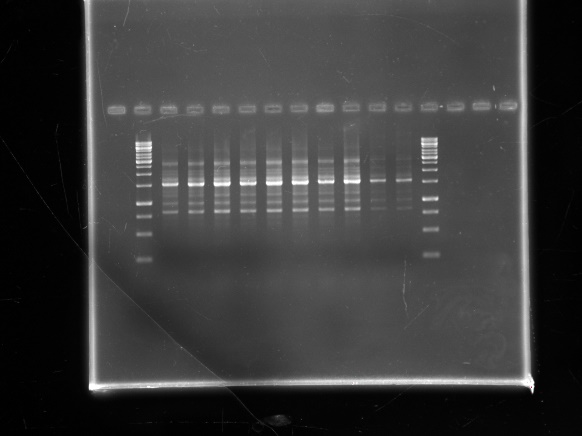


SCoT 16: L: Ladder, M: Mother plant, 1-9: In vitro regenerated plants


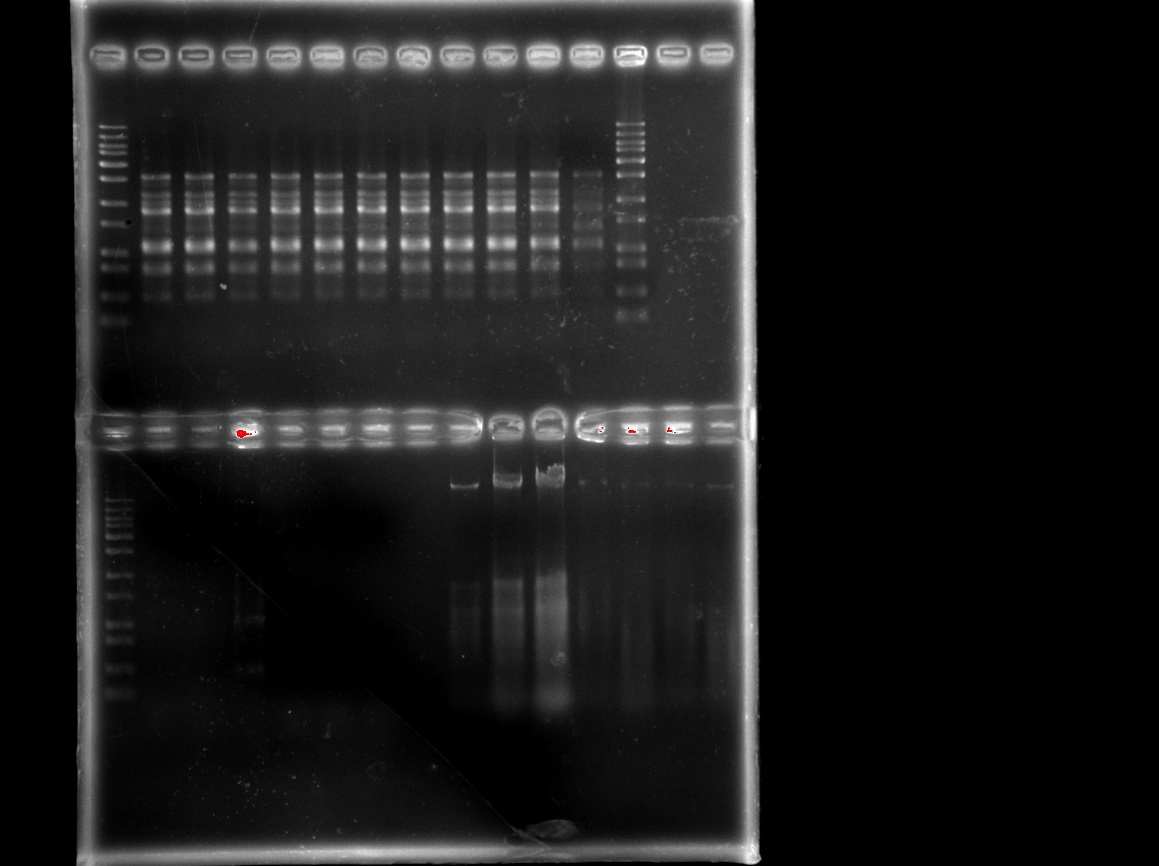

Supplement: Supplementary file 1 — Additional file 1: Supplementary data. SCoT 9: L: Ladder, M: Mother plant, 1–9: In vitro regenerated plants. SCoT 16: L: Ladder, M: Mother plant, 1–9: In vitro regenerated plants. [file 43141_2023_523_MOESM1_ESM.docx]
